# GABA and Octopamine Receptors as Potential Targets for Fumigant Actions of *Bursera graveolens* Essential Oil Against *Callosobruchus maculatus* and *Callosobruchus chinensis*

**DOI:** 10.3390/jox15030091

**Published:** 2025-06-12

**Authors:** Luis O. Viteri, Maria José González, Pedro B. Silva, Jonatas M. Gomes, Thiago Svacina, Lara T. M. Costa, Eduardo Valarezo, Javier G. Mantilla-Afanador, Osmany M. Herrera, Raimundo W. S. Aguiar, Gil R. Santos, Eugênio E. Oliveira

**Affiliations:** 1 Programa de Pós-Graduação em Produção Vegetal, Universidade Federal do Tocantins, Gurupi 77402-970, TO, Brazil; osmany.herrera@uft.edu.br (O.M.H.); gilrsan@uft.edu.br (G.R.S.); 2 Programa de Pós-Graduação em Biologia Animal, Universidade Federal de Viçosa, Viçosa 36570-900, MG, Brazil; 3 Programa de Pós-Graduação em Biotecnologia, Universidade Federal do Tocantins, Gurupi 77402-970, TO, Brazil; rwsa@mail.uft.edu.br; 4 Departamento de Bioquímica e Imunologia, Universidade Federal de Minas Gerais, Belo Horizonte 31270-901, MG, Brazil; majito130692@ufmg.br; 5 Departamento de Entomologia, Universidade Federal de Viçosa, Viçosa 36570-900, MG, Brazil; pedrobento1709@gmail.com (P.B.S.); jonatas.gomes@ufv.br (J.M.G.); svacina@usp.br (T.S.); lara.t.costa@ufv.br (L.T.M.C.); 6 Departamento de Química, Universidad Técnica Particular de Loja, Loja 1101608, Ecuador; bevalarezo@utpl.edu.ec; 7 Grupo de Pesquisa em Microbiologia e Biotecnologia Agroindustrial, Universidad Católica de Manizales, Road 23 N. 60, Manizales 170002, Colombia; jmantilla@ucm.edu.co

**Keywords:** *Callosobruchus* spp., cowpea, weevils, eco-friendly, essential oils, GABA, octopamine, target site

## Abstract

Cowpea, *Vigna* sp., is an important, low-cost protein source in subtropical and semi-arid regions, where seasonal rainfall makes storage necessary. However, the weevils *Callosobruchus maculatus* and *C. chinensis* cause significant grain losses during storage. While synthetic fumigants are commonly used to control these pests, their risks to mammals have prompted the search for safer alternatives. In this context, we tested palo santo, *Bursera graveolens*, essential oil with limonene, *α*-phellandrene, *o*-cymene and *β*-phellandrene, menthofuran, and germacrene-D as a sustainable approach. This plant is readily accessible, produces high fruit yields, and is used in households for various purposes. We evaluated the fumigant toxicity, repellency, and ovicidal effects of *B. graveolens* essential oil on both *Callosobruchus* species. Our results showed that *B. graveolens* oil was toxic to *C. maculatus* (LC_50_ = 80.90 [76.91–85.10] µL) and *C. chinensis* (LC_50_ = 63.9 [60.95–66.99] µL), with *C. chinensis* being more susceptible (SR = 1.27). Molecular docking analyses revealed that all the oil’s compounds bind to both the GABA and octopamine receptors, exhibiting high energy affinities; however, germacrene shows the strongest affinity in these receptors. *C. chinensis* was strongly repelled at all concentrations, while *C. maculatus* was repelled only at lethal concentrations. No ovicidal effect was observed in either species. In conclusion, our findings suggest that *B. graveolens* essential oil is a promising and sustainable protectant for stored cowpeas in small-scale storage units.

## 1. Introduction

Cowpea *Vigna genus* (L.) is a staple food in semi-arid tropical regions due to its high-quality nutritional content and low cost compared to other protein sources [1,2]. In 2022, global production exceeded 9.77 million metric tons [3]. Like all grains, cowpeas must be stored for later consumption, which can lead to infestations by adapted insects. The primary pests of cowpea across the five continents are the cosmopolitan weevils *Callosobruchus maculatus* Fabricius and *Callosobruchus chinensis* L. [4,5]. Infestation typically begins in the field, as females lay eggs in open bean pods, and continues in storage. The rapid reproduction and high fertility of *C. maculatus* and *C. chinensis,* with a new generation emerging in less than 30 days and a mean fertility of 100 eggs per female [6,7] make effective management essential in storage units. Conversely, these infestations can result in losses exceeding 30% within a few months [8].

The most common method for controlling stored pests is phosphine-based fumigants. While these fumigants are highly effective, relatively inexpensive, and food-safe, they have some drawbacks, including corrosive effects on metals, environmental pollution [9], and the development of resistance [10,11,12]. As a result, research into alternative pest control methods has increased, with a focus on essential oils and their bioactive compounds. Essential oils are a complex mixture of molecules with potential biological effects, including acute and chronic toxicity [13,14], repellent activity [15,16], and the inhibition of oviposition, growth, feeding, and the development of organisms [17,18,19,20]. This wide range of possible actions of essential oils is due to their diverse chemical composition, in which the effects on organisms can respond to the main compounds or the interaction between various compounds. Additionally, these products minimize harm to non-target organisms such as pollinators, natural enemies, and mammals [21,22].

The complex composition of essential oils makes it difficult for insects to develop resistance to these products. However, it also complicates the understanding of the specific sites of action on target organisms. Recent advances in bioinformatics have identified potential target sites for isolated compounds in essential oils. For example, it is known that the primary physiological targets of essential oils are specific proteins involved in insect metabolism or key neurotransmitters that are inhibited or altered [21,23]. Studies have identified several targets, including gamma-aminobutyric acid (GABA) receptors, transient receptor potential (TRP) channels, octopamine receptor (OctpR), acetylcholine esterase (AChE), enzymatic [superoxide dismutase (SOD), catalase (CAT), peroxidases (POx), glutathione-S-transferase (GST) and glutathione reductase (GR)], and non-enzymatic [glutathione (GSH)] antioxidant defense systems [23,24,25,26].

Thus, *Bursera graveolens* (Burseraceae), commonly called palo santo, could serve as a valuable source of biomolecules with insecticide potential, as it is rich in monoterpenes and sesquiterpenes [27,28]. Additionally, since the fruit is used to extract essential oil with a yield of over >3%, its use is both profitable and sustainable. The biological properties of *B. graveolens* include insecticide, acaricide, and antimicrobial effects, as well as medicinal benefits [29,30,31,32,33]. This study evaluates the fumigant effect of *B. graveolens* essential oil on *C. maculatus* and *C. chinensis*, the two primary pests of stored cowpeas. Additionally, 3D molecular modeling and in silico molecular docking were used to investigate the binding patterns of these compounds to target sites in the insects.

## 2. Materials and Methods

### 2.1. Rearing Insects Callosobruchus maculatus and C. chinensis

The original populations of *C. maculatus* and *C. chinensis*, the main pests of cowpea bean (genus *Vigna*), were field-collected from small farms in the Viçosa region (Minas Gerais State, Brazil—20°45′20.23″ S, 42°51′50.95″ W) during the years 2018–2019. Both species were reared on their primary host, free from insecticide residues and other infestations. Cowpea (*V. unguiculate*) was obtained from the local market, and the grains were stored at −10 °C for two weeks before being provided to the weevils, ensuring the grains were free from other insect contamination. The adults were placed in ventilated glass jars (4 L) with the beans and maintained under laboratory conditions (27 ± 2 °C, 75 ± 5% relative humidity, 12 h of scotophase) [19].

### 2.2. Bursera graveolens Essential Extraction Oil

The essential oil (EO) of *B. graveolens* was extracted from fresh fruits using the hydrodistillation method with a Clevenger-type apparatus at approximately 100 °C for three hours. The EO was then dried over anhydrous sodium sulfate and stored in sealed vials protected from light at 4 °C until further analysis and use [29]. Chromatographic analysis using gas-chromatography mass spectrometry (GC-MS) with a gas chromatograph SHIMADZU GCMS-QP500, (Shimadzu Corporation, Kyoto, Japan) equipped with a mass detector by electron impact ionization (70 eV) and a GC-2014 (SHIMADZU) gas chromatograph equipped with a flame ionization detector (GC-FID), revealed that the *B. graveolens* essential oil used here has aliphatic monoterpenes limonene (43.6%), α-phellandrene (20.4%), o-cymene (12.7%), and β-phellandrene (3.7%) as major compounds; the oxygenated monoterpene menthofuran (10.7%) and the aliphatic sesquiterpene germacrene D (2.1%) [16].

### 2.3. Bioassays Toxicity Against Callosobruchus maculatus and Callosobruchus chinensis

The fumigant effect of the essential oil was tested on two species of weevils in cowpea beans and their primary host, following standardized methods described by Ndomo et al. [34] and Zapata and Smagghe [35]. Briefly, the experiment involved a 250 cm^3^ glass jar containing 75 g of cowpea grains and 20 newly emerged adult insects (<24 h) and sexed (1:1 male-to-female ratio). Several concentrations [40, 60, 80, 140 µL/L (*C. chinensis*) and 40, 60, 80, 100, 180, 220 µL/L (*C. maculatus*)] of *B. graveolens* essential oil were used. Each concentration was applied in a small 4 cm^2^ piece of Whatman No. 1 filter paper that was attached to the metal lid with cotton thread and suspended inside the jar without touching the beans, acting as a diffuser. The jars were then sealed with parafilm (PM996, Parafilm, Detroit, MI, USA) and placed in a BOD incubator with constant conditions of temperature (27 ± 2 °C), relative humidity (70 ± 5%), and 12 h scotophase. After 24 h, the dead insects in each jar were counted and considered dead if they did not respond to a contact stimulus applied with a fine brush. Five replicates were conducted for each treatment. The control group followed the same experimental setup, but without the application of essential oil.

### 2.4. Molecular Docking Between Essential Oil Biomolecules of Bursera graveolens and Gamma-Aminobutyric Acid (GABA) and Octopamine Receptors of Callosobruchus maculatus

The proteome of *C. maculatus* (cowpea weevil) is available from the National Center for Biotechnology Information (NCBI) database. We performed a BLASTp of *C. maculatus* proteome search against the (NCBI) databases and identified the protein gamma-aminobutyric acid receptor (GABA) subunit beta XP_050512277.1 of *Diabrotica virgifera virgifera* (Coleoptera; Chrysomelidae) with 90% identity and an e-value of 0.0, matching with the VEN59736.1 unnamed protein (*C. maculatus*). We downloaded the VEN59736.1 amino acid sequences of *C. maculatus* and used the method with Swiss Model Workspace https://swissmodel.expasy.org/interactive (15 January 2025) in order to build the protein by inspecting protein structure crashes, the amino acid positions at the active site [36], Ramachandran plots [37], and the Global Model Quality Estimate (GMQE). The prediction was based on the structure generated using AlphaFold DB [38] for the protein identified as A0A653DHQ0 (Uniprot ID: A0A653DHQ0_CALMS). The resulting model exhibited a typical conformational architecture of GABA receptors, with well-defined transmembrane domains. The model quality assessment using GMQE of (0.77) and the values of Ramachandran, favored with 90%, indicated a reliable confidence level in the prediction. In the BLASTp search for an octopamine receptor of the *C. maculatus* proteome, using the (NCBI) databases, we identified the protein octopamine receptor beta-3R-like XP_023024593.1 of *Leptinotarsa decemlineata* (Coleoptera; Chrysomelidae), with 83.4% of identity and e-value 3 × 10^−163^ matching with the VEN45923.1 unnamed protein (*C. maculatus*). The prediction was based on the structure generated using AlphaFold DB for the protein identified as A0A6P7F675 (Uniprot ID: A0A6P7F675_DIAVI). The resulting model exhibits the typical architecture **of G-protein-coupled receptors (GPCRs),** with well-defined transmembrane domains, consistent with its role in neurotransmission. The model quality assessment using a GMQE of 0.83 and values of Ramachandran of 95.5% indicated a reliable confidence level in the prediction. For both modeled proteins, GABA and octopamine, the energy minimization was performed using the Yasara forced field [39].

We prepared the limonene, *α*-phellandrene, *o*-cymene, menthofuran, *β*-phellandrene, and germacrene D molecules of *B. graveolens* using PubChem [40] and NCBI and stored them in a Structure Data Format (SDF) for molecular docking predictions. These molecules and proteins were prepared with Autodock Tools 1.5.7. [41]. The best ligand–receptor complex, which returned affinity energy values (kcal/mol) using the AutoDock Vina [42] was used to generate 2D interaction maps with Discovery Studio Visualizer.

### 2.5. Repellency Bioassay of Essential Oil Against Callosobruchus *spp.*

**Figure 1:**
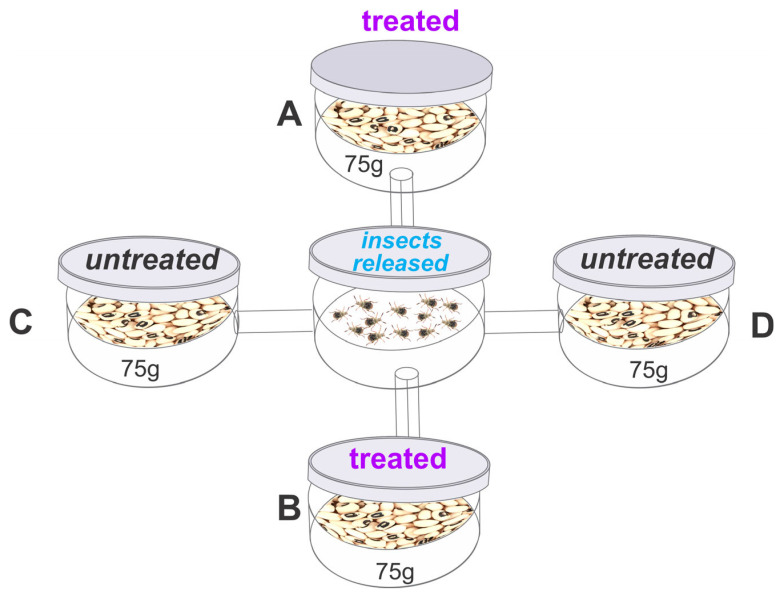
Layout of the arena used in the repellency bioassay; The arenas A, B with *Bursera graveolens* essential oil and arenas C, D without treatment.

The bioassay to assess the repellent activity of the essential oil was conducted using an apparatus consisting of five circular plastic containers (12 cm diameter, 8 cm height). A central container (E) was connected to four surrounding containers (A, B, C, and D) using plastic cylinders (12 cm long, 1 cm in diameter) (Figure 1)**,** as described by Jumbo, Corrêa, Gomes, Armijos, Valarezo, Mantilla-Afanador, Machado, Rocha, Aguiar, and Oliveira [16], Freitas et al. [43]. Containers A and B were arranged diagonally and filled with 75 g of cowpea beans treated with an estimated sublethal or lethal concentration of *B. graveolens* essential oil [(in μL/L): LC_15_ or LC_50_ or LC_95_)] for each species. The essential oil was applied to filter paper hanging from the lid of the container, as described in the toxicity bioassay. Containers C and D were filled with 75 g of untreated beans (control). In the central container, 40 unsexed adults (<24 h old) were released, and after 24 h, the total number of insects in each container was recorded. The repellency index (RI) was calculated as proposed by Mazzonetto and Vendramim [44] RI = (2*T*)/(*T* + *C*), where RI is the repellency index, *C* is the percentage of insects in the untreated containers, and *T* is the percentage of insects in the treated containers. The RI values ranged from 0 to 2, with the following interpretation: RI = 1 (neutral activity), RI > 1 (attraction), and RI < 1 (repellence). A safety margin for classification was established by adding or subtracting the standard deviation (SD) of each treatment from a value of 1 (indicating neutrality).

### 2.6. Tests Effect Ovicidal and Emergency Adults Callosobruchus *spp.*

To determine the ovicidal activity of the essential oil, the methodology of Nattudurai et al. [45] was adapted. Glass jars, as described in the toxicity assay process, were used for each species. Twenty insects (10 males and 10 females) of *C. maculatus* or *C. chinensis* were placed in 20 g of cowpea bean for egg laying. After 48 h, the beetles were removed, and the beans containing eggs were exposed to four estimated concentrations (LC_25_, LC_50_, LC_75_, LC_95_) of *B. graveolens* essential oil for 24 h, as performed in the toxicity assay. After five days, the number of larvae that hatched from the eggs was counted. The beans from each exposure were transferred to new containers, and the progeny of the adult of each species and treatment were assessed after 35 days [19]. Four replicates were set up for each concentration of essential oil and the control.

### 2.7. Statistical Analysis

Concentration–mortality curves were estimated using probit analyses with the PROC PROBIT procedure in SAS software, version 9.1 [46]. The 95% confidence intervals for susceptibility rate (SR) were calculated as described by Robertson et al. [47], with values considered significant if the range did not include 1. The number of hatched larvae in each treatment was compared to control emergence using the Bonferroni test (*p* < 0.05) and GraphPad Prisma 8.1.

## 3. Results

### 3.1. Essential Oil Toxicity Bioassays to Callosobruchus maculatus and Callosobruchus chinensis

**Figure 2:**
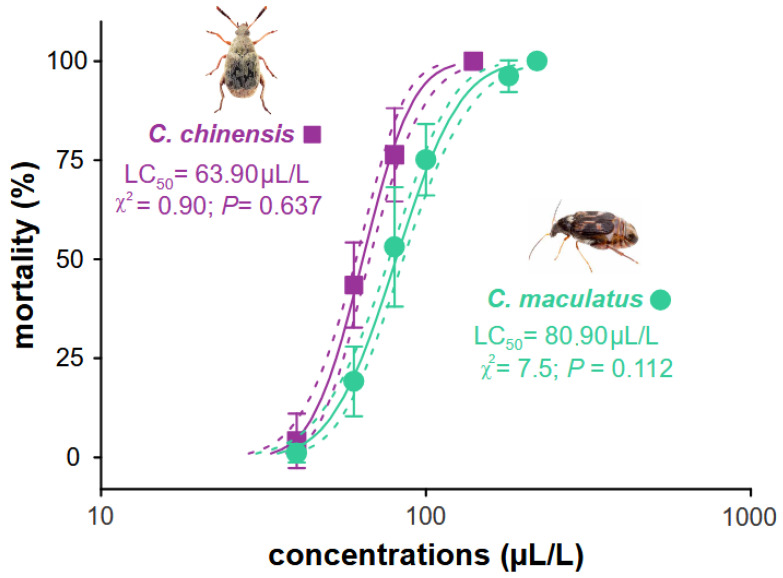
Concentration–mortality curve estimated for *Bursera graveolens* essential oil for *Callosobruchus maculatus* and *C. chinensis*. The full lines represent the estimated lethal concentration (LC) of each species, and the dotted lines represent the confidence intervals (95%). Symbols represent the average (±SE) mortality recorded for each essential oil concentration of four replicates.

Our results showed that *B. graveolens* essential oil is toxic to the two main pests of stored cowpea beans when used as a fumigant. The lethal concentration (LC_50_) was estimated to be *C. maculatus* LC_50_ 80.90 (76.91–85.10) µL, which was greater than for *C. chinensis* 63.90 (60.95–66.99) µL, with a susceptibility ratio (SR) of 1.27 for *C. maculatus* (Table 1). These values for each species were obtained from the concentration–mortality curves from the probit model (goodness-of-fit tests exhibited low χ^2^ values and high *p*-values [>0.05] when all the concentrations for each species were obtained (Figure 2)).

### 3.2. Interactions Between Bursera graveolens Essential Oil and the Site’s Target of Callosobruchus *spp.*

#### 3.2.1. *Bursera graveolens* Essential Oil Interaction with Gamma-Aminobutyric Acid (GABA)

**Figure 3:**
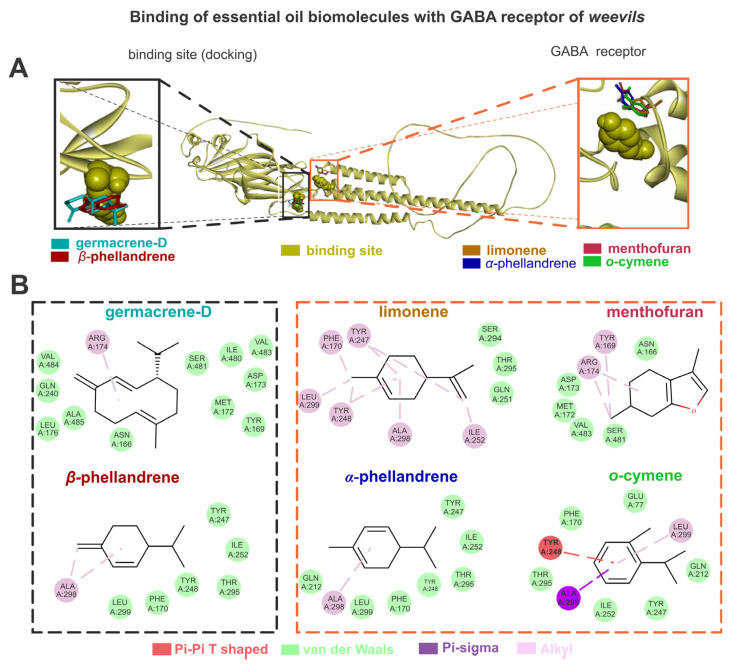
The limonene, o-cymene, α-phellandrene, β-phellandrene, germacrene D, and menthofuran bind with gamma-aminobutyric acid (GABA) sites of weevils (**A**); the molecular interactions of the same constituents with the amino acids from GABA active sites are also shown (**B**).

The GABA exhibited higher energy affinities (AutoDockVina affinity energy kcal mol^–1^) when complexed with germacrene D (–6.3), followed by limonene (–5.1), α-phellandrene (–5.0), o-cymene (–5.0), menthofuran (–5.0), and β-phellandrene (–4.9). The germacrene D showed van der Waals interactions with LEU176, ALA485, GLN240, GLU233, VAL484, ASN166, TYR169, MET172, ASP173, SER481, ILE480, and VAL483, as well as alkyl interactions with ARG174. The limonene showed van der Waals interactions with THR295, SER294, and GLN251, as well as alkyl interactions with TYR247, LEU299, TYR248, PHE170, ALA298, and ILE252. The α-phellandrene showed van der Waals interactions with TYR247, ILE252, THR295, TYR248, PHE170, LEU299, and GLN212, as well as alkyl interactions with ALA298. The o-cymene showed van der Waals interactions with ILE252, TYR247, GLN212, GLU77, PHE170, and THR295, pi-sigma interactions with ALA298, pi-pi T-shaped interactions with TYR248, and alkyl interactions with LEU299. The menthofuran showed van der Waals interactions with ASN166, SER481.VAL483, MET172, and ASP173, as well as alkyl interactions with TYR169 and ARG174. The β-phellandrene showed van der Waals interactions with TYR247, ILE252, THR295, TYR248, PHE170, and LEU299, as well as alkyl interactions with ALA298 (Figure 3).

#### 3.2.2. *Bursera graveolens* Essential Oil Interaction with Octopamine Receptors

**Figure 4:**
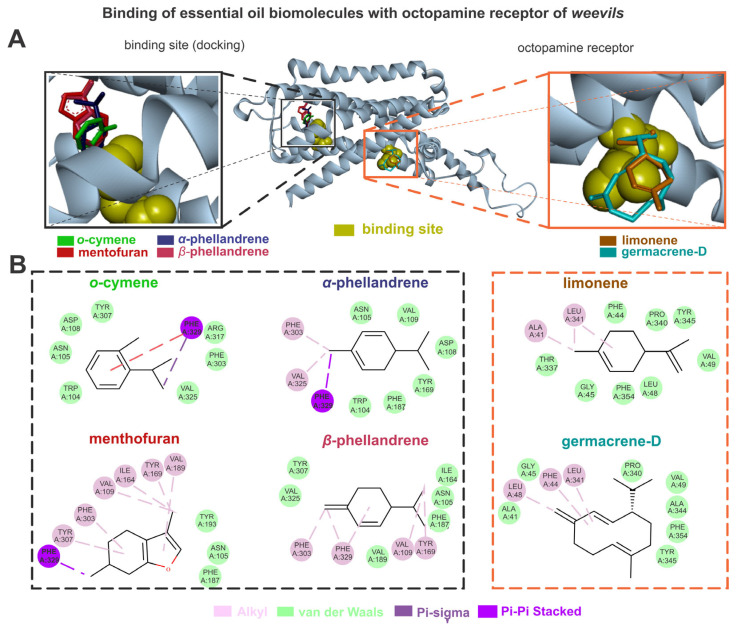
The limonene, o-cymene, α-phellandrene, β-phellandrene, germacrene D, and menthofuran bind with octopamine sites of weevils (**A**); the molecular interactions of the same constituents with the amino acids from octopamine active sites are also shown (**B**).

The biomolecules of the *B. graveolens* and octopamine receptors exhibited higher energy affinities when complexed with germacrene D (–6.7), menthofuran (–6.1), *α*-phellandrene and *β*-phellandrene (5.8), *o*-cymene (–5.7), and limonene (–5.4). The germacrene D showed van der Waals interactions with TYR345, PHE354, ALA344, VAL49, PRO340, GLY45, and ALA41, and alkyl interactions with LEU341, LEU48, and PHE44. The menthofuran showed van der Waals interactions with TYR193, ASN105, and PHE187, alkyl interactions with TYR307, PHE303, VAL109, ILE164, TYR169, and VAL189, and pi-sigma interactions with PHE329. The *α*-phellandrene showed van der Waals interactions with PHE187, TRP104, TYR169, ASP108, VAL109, and ASN105, as well as alkyl interactions with VAL325 and PHE303, and pi-sigma interactions with PHE329. The *β*-phellandrene showed van der Waals interactions with ILE164, ASN105, PHE187, VAL189, TYR307, VAL325, and VAL189, as well as alkyl interactions with TYR169, VAL109, PHE329, and PHE303. The *o*-cymene showed van der Waals interactions with VAL325, PHE303, ARG317, TYR307, ASP108, ASN105, and TRP104, pi-sigma interactions with PHE329, and pi-pi stacked interactions with PHE329. The limonene showed van der Waals interactions with PHE44, PRO340, TYR345, VAL49, LEU48, PHE354, GLY45, and THR337, as well as alkyl interactions with LEU341 and ALA41 (Figure 4).

### 3.3. Repellency of Bursera graveolens Essential Oil to Adult Callosobruchus maculatus and Callosobruchus chinensis

**Figure 5:**
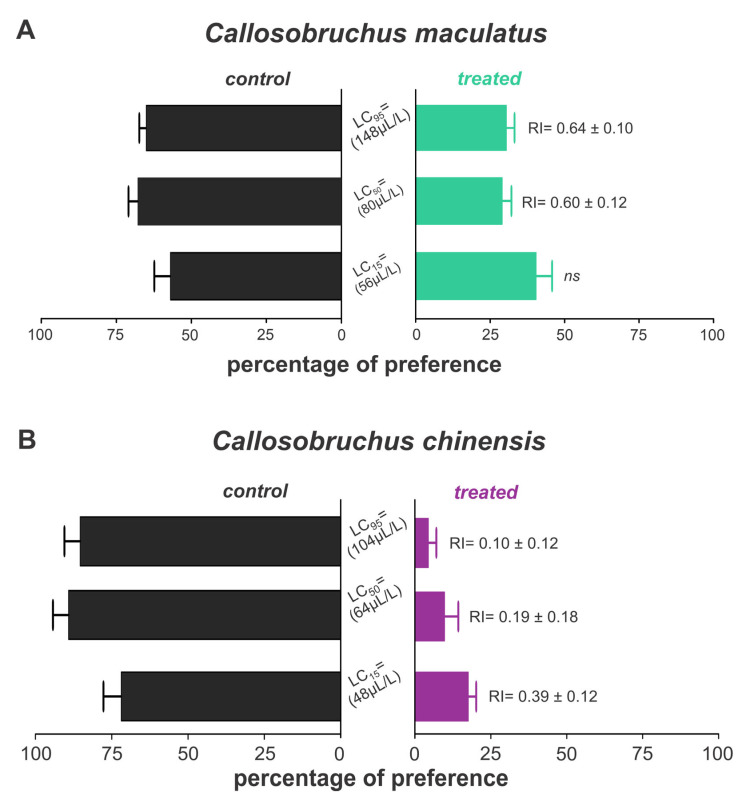
Repellency of *Bursera graveolens* essential oil to adult *Callosobruchus maculatus* (**A**) and *Callosobruchus chinensis* (**B**) in cowpea beans. Bars represent the average (±SE) percentage of insects found after 24 h of exposure.

The essential oils of *B. graveolens* exhibited repellent effects on both weevil species, with variations depending on species and concentration. Thus, *C. maculatus* was repelled when exposed only to lethal concentrations, with approximately 30% of insects in the treated grains (Figure 5A). On the other hand, we found 4.47, 9.78, and 17.61% of *C. chinensis* on the grains treated with LC_15_, LC_25,_ and LC_50,_ respectively, indicating a strong repellent activity against this species (Figure 5B).

### 3.4. Ovicidal Effect of Bursera graveolens Essential Oil

The early-age eggs (24 h) from *C. chinensis* and *C. maculatus* were not susceptible to B. graveolens essential oil when applied as a fumigant. In both species, the emergence rate was between 50 and 70%, approximately, and no difference was found from the control in *C. chinensis* (F_4,19_ = 2.28; *p* = 0.108) and *C. maculatus* (F_4,19_ = 2.25; *p* = 0.111) (Table 2).

## 4. Discussion

Our results suggest that *B. graveolens* essential oil may be effective in controlling two major cowpea storage pests, *C. maculatus* and *C. chinensis*. In silico analysis indicates that all compounds in *B. graveolens* essential oil (germacrene, limonene, *α*-phellandrene, menthofuran, *β*-phellandrene, and *o*-cymene) interact with both γ-aminobutyric acid (GABA) and octopamine receptors found in the weevils’ nervous system, with the sesquiterpene germacrene showing the strongest affinity. While no ovicidal effects were observed, the oil shows promise as both a curative treatment due to its toxicity and a preventive measure because of its repellent properties.

The *B. graveolens* essential oil was toxic to both *Callosobruchus* species, although the susceptibility rates differed. *C. chinensis* was more susceptible than *C. maculatus*. Similar susceptibility of *C. chinensis* to other biocontrol methods has been reported, including ozone [5], essential oils [48], microorganisms [49] and synthetic insecticides [50]. The differences in insect susceptibility to a particular compound can mainly be attributed to species-specific detoxification strategies when exposed to xenobiotics. Gupta et al. [51] demonstrated variation in detoxification activity (acetylcholine esterase and glutathione-S-transferase) in these same species when exposed to the same essential oil. Additionally, the toxicity of *B. graveolens* to weevils was linked to its major compounds, though the synergic effect of minor components should not be overlooked. Previous studies have highlighted the effectiveness of *B. graveolens* essential oil against stored-grain pests and other arthropods [16,29,33,52], as well as the toxicity of major compounds like limonene [53] or phellandrene [54,55] via contact or fumigant methods. However, due to the complexity of essential oils, which contain compounds with additive, antagonistic, or synergistic effects, the exact mode of action on target organisms remains uncertain. Therefore, individual analyses of the major compounds are necessary.

In silico molecular docking analysis revealed that the main compounds of *B. graveolens*, such as limonene, *α*-phellandrene, *o*-cymene, menthofuran, *β*-phellandrene, and germacrene, showed the highest binding affinity for both γ-aminobutyric acid (GABA) and octopamine receptors. However, the germacrene compound demonstrated greater structural stability in both receptors, enhancing hydrophobic interactions. The germacrene-GABA receptor complex exhibited stronger van der Waals interactions, indicating more contact points and increased stability with the GABA protein. This high affinity was also evident with the octopamine receptor, contributing to a strong hydrophobic complementarity with the protein [56]. Given that y-aminobutyric acid (GABA) receptors are critical for inhibitory neurotransmission in insects, and octopamine receptors serve as neurotransmitters, neuromodulators, or neurohormones in both central and peripheral nervous systems, even small changes in these receptors can trigger significant physiological events [57,58]. GABA receptors in insects are known targets of synthetic insecticides, classified as Group 2 molecules by IRAC [59]. This group is represented by phenylpyrazoles, cyclodienes, organochlorines, meta-diamides, and isoxazolines [60,61], while octopamine receptors are targeted by antagonists like amitraz (IRAC Group 19) and new molecules with potential insecticide such as Phenyl imidazolinium-2-one [62,63,64]. Synergism effects in insecticides targeting GABA or octopamine receptors have been documented [65,66], suggesting that similar synergistic interactions may occur with the organic compounds in essential oil. Essential oils have been reported to act on octopaminergic receptors in insects as agonists and/or antagonists, inducing intracellular changes in cAMP production [67,68]. Additionally, *B. graveolens* essential oils have been shown to disrupt the functions of acetylcholinesterase (AChE) enzymes and transient receptor potential (TRP) channels in insects [16]. Our in silico analyses support GABA and octopamine receptors as potential targets, mainly for germacrene.

Studies have shown that essential oils containing the sesquiterpene germacrene as a major compound are toxic and deterrent to insects, including stored-grain pests [69,70,71]. Although limonene and phellandrene have a lower affinity for GABA and octopamine receptors compared to germacrene, these compounds are predominant in the essential oil and likely contribute significantly to its toxicity. The toxicity of essential oils containing limonene or phellandrene, as well as pure compounds, has been demonstrated [72,73]. Recent studies have shown that essential oils rich in limonene and phellandrene inhibit enzymes such as glutathione-S-transferase, acetylcholinesterase, and Na^+^/K^+^-ATPase in *C. chinensis* and *C. maculatus,* among other insects [73,74,75]. Additionally, germacrene has been shown to increase Reactive Oxygen Species (ROS) and disrupt antioxidant defense systems in these species [76], as well as cause damage to other insects’ organs [77,78].

As a preventive measure against *C. maculatus* and *C. chinensis* colonization in stored cowpea beans, *B. graveolens* essential oil proved effective due to its repellent effect. While higher concentrations were more repellent to *C. maculatus*, *C. chinensis* was strongly repelled even at lower concentrations. Similar findings were reported by Jayaram et al. [79] with *Tagetes minuta* essential oil, which showed stronger deterrence for *C. chinensis*. However, it is already known that the repellent effect varies by organism type and/or concentration applied [14,80]. The repellent properties of *B. graveolens* essential oil against stored-grain weevils, including the common bean weevil, have been documented [16,31,33] with monoterpenes like limonene and phellandrene, primarily responsible for these effects. The repellent activity of these compounds was also observed by Cao, Pang, Guo, Wang, Geng, Sang, Guo, and Du [54], supporting our hypothesis, which was further confirmed through electroantennography studies showing insect olfactory responses to limonene [81,82]. Additionally, the germacrene compound, which exhibited a high affinity for GABA and octopamine receptors, also demonstrated strong repellent effects against other insects [83,84].

The *B. graveolens* essential oil was toxic and repellent to *C. maculatus* and *C. chinensis*; however, no ovicidal effects were observed at any of the applied concentrations. Previous studies on the fumigant effect of essential oils on *Callosobruchus* species eggs have yielded mixed results, with some reporting no effects, while others found 100% ovicidal activity on 24-hour-old eggs [45,85,86,87]. The effectiveness against eggs depends on the essential oil composition, concentration, and egg age, as demonstrated by *Acanthoscelides obtectus* (Say 1831) [88,89]. Based on our results, *B. graveolens* may be less effective at controlling these beetles when the grains are infested with early-stage eggs or may require prolonged exposure to affect embryo development.

## 5. Conclusions

Our results demonstrate the preventive and curative potential of *Bursera graveolens* essential oil against the two primary pests of stored cowpeas, *Callosobruchus maculatus* and *C. chinensis*. Given that the oil is extracted from the tree’s fruit with a high yield, it could provide a promising and sustainable solution for controlling these pests in tropical regions, especially in small-scale farming, where cowpea beans are widely cultivated. However, further research is needed to stabilize the product and conduct selectivity tests on mammals.

## Figures and Tables

**Table 1:** Toxicity of *Bursera graveolens* essential oil against the primary pests of stored cowpea, *Callosobruchus chinensis* and *Callosobruchus maculatus*, when exposed as a fumigant for 24 h.

Essential Oil	Weevil Species	N°	Slope ± SD	Estimated LC (µL/L) 95% CI	χ^2^	*p*-Value	SR (95%CI)
*Bursera graveolens*	*Callosobruchus* *maculatus*	620	6.38 ± 1.04	LC_10_ = 50.96 (46.35–54.93)	7.50	0.11	1.27 (1.24–1.29) a
				LC_50_ = 80.90 (76.91–85.10)			
				LC_75_ = 103.16 (97.42–110.41)			
				LC_95_ = 146.35 (133.84–164.25)			
	*Callosobruchus* *Chinensis*	402	8.23 ± 0.78	LC_10_ = 44.63 (40.54–47.51)	0.90	0.64	1.00 (0.98–1.02) b
				LC_50_ = 63.90 (60.95–66.99)			
				LC75 = 77.17 (73.20–82.47)			
				LC_95_ = 101.25 (93.10–113.81)			

LC (lethal concentration); SR (susceptibility ratio) [47]. Different letters in the Susceptibility Rate (SR) signified difference based in the Confidence Interval (95%).

**Table 2:** Percentage of egg hatching in *Callosobruchus maculatus* and *Callosobruchus chinensis* exposed at an early age (24 h) to estimated concentrations of *Bursera graveolens* essential oil.

Concentration (µL)	*Callosobruchus maculatus*	*Callosobruchus chinensis*
Control	71.24 ± 5.16 a	63.47 ± 11.64 a
LC_25_	68.80 ± 3.41 a	69.70 ± 3.40 a
LC_50_	65.28 ± 1.92 a	59.29 ± 3.17 a
LC_75_	67.89 ± 6.69 a	59.62 ± 5.30 a
LC_95_	57.11 ± 13.88 a	57.43 ± 5.41 a

Same letters in the same column indicate no statistically significant difference between the control and each concentration, as determined by the Bonferroni test (*p* < 0.05).

## Data Availability

No new data were created or analyzed in this study. Data sharing is not applicable to this article.
